# Selective cultivation of bacterial strains with lipolytic and hydrocarbon-oxidizing activity from bottom sediments of the Ob River, Western Siberia

**DOI:** 10.18699/VJGB-22-55

**Published:** 2022-08

**Authors:** A.L. Gerasimchuk, D.A. Ivasenko, A.A. Kasymova, Yu.A. Frank

**Affiliations:** Tomsk State University, Tomsk, Russia; Tomsk State University, Tomsk, Russia Darvin LLC, Tomsk, Russia; Tomsk State University, Tomsk, Russia; Tomsk State University, Tomsk, Russia Darvin LLC, Tomsk, Russia

**Keywords:** microorganisms-decomposers, phylogenetic diversity, producers, lipolytic activity, organic substrates, biotechnological potential, микроорганизмы-деструкторы, филогенетическое разнообразие, продуценты, липолитическая активность, органические субстраты, биотехнологический потенциал

## Abstract

Bacteria play a key role in biogeochemical cycles in natural and anthropogenic ecosystems. In river ecosystems, bacteria intensively colonize silt sediments. Microorganisms are essential for energy conversion, biogeochemical nutrient cycling, pollutant degradation, and biotransformation of organic matter; therefore, bottom sediments can be a source of metabolically diverse microorganisms, including those with promise for industrial biotechnologies. The aim of this work was to isolate and study pure cultures of microorganisms – producers of industrially important enzymes and decomposers of organic matter – from bottom sediments of the Ob River. Pork fat and diesel fuel were used as substrates to obtain enrichment and pure cultures for selective cultivation of bacteria with lipolytic and hydrocarbon-oxidizing activity. A total of 21 pure cultures were isolated. The phylogenetic position of the obtained bacterial isolates was determined based on the analysis of 16S rRNA gene sequences. The strains isolated on selective media belonged to representatives of the genera Pseudomonas and Aeromonas (Gammaproteobacteria), and the genus Microvirgula (Betaproteobacteria). The ability of strains to grow on culture media containing pork fat, olive oil and diesel fuel was analyzed. The lipolytic activity of the isolates was evidenced by cultivation on a diagnostic medium containing 1 % tributyrin. The phylogenetic and metabolic diversity of the cultivated non-pathogenic bacterial strains with lipolytic and oil-oxidizing activity revealed in the study indicates the biotechnological potential of the isolates. The most promising strains were M. aerodenitrificans sp. LM1 and P. lini sp. KGS5K3, which not only exhibited lipolytic activity on the diagnostic medium with tributyrin in a wide temperature range, but also utilized diesel fuel, pork fat and olive oil.

## Abstract (ru)

Бактерии играют ключевую роль в биогеохимических циклах природных и антропогенных экосистем. В речных экосистемах бактерии, как правило, интенсивно заселяют илистые отложения. Микроорганизмы имеют важное значение в преобразовании энергии и биотрансформации органических веществ. В связи с этим донные отложения, богатые органикой, могут являться источником выделения метаболически разнообразных микроорганизмов, в том числе перспективных для промышленных биотехнологий. Целью данного исследования было выделение и изучение чистых культур микроорганизмов – продуцентов промышленно значимых ферментов и деструкторов органических веществ из донных осадков р. Оби. В качестве субстратов для выделения накопительных и чистых культур использовали свиной жир и дизельное топливо для селективного культивирования бактерий с липолитической и углеводородокисляющей активностью. Всего получена 21 чистая культура. Филогенетическое положение бактериальных изолятов определено на основе анализа последовательностей генов 16S рРНК. Выделенные на селективных средах штаммы оказались представителями родов Pseudomonas и Aeromonas класса Gammaproteobacteria и рода Microvirgula класса Betaproteobacteria. Изучена способность штаммов к росту на плотных питательных средах со свиным жиром, оливковым маслом и дизельным топливом. Липолитическая активность штаммов подтверждена культивированием на диагностической среде с трибутирином. Обнаруженное в ходе исследований филогенетическое и метаболическое разнообра- зие культивируемых непатогенных бактериальных штаммов с липолитической и нефтеокисляющей активно- стью указывает на биотехнологический потенциал выделенных нами изолятов. Наиболее перспективными оказались штаммы M. aerodenitrificans sp. LM1 и P. lini sp. KGS5K3, которые не только проявили липолитическую активность на диагностической среде с трибутирином в широком диапазоне температур, но и утилизировали такие сложные органические субстраты, как дизельное топливо, свиной жир и оливковое масло.

## Introduction

Bacteria play a significant role in the biogeochemical cycles in natural and anthropogenic ecosystems. In river ecosystems, bacteria intensively colonize silt sediments (Araya et al., 2003). The river microbial network is a directional linear branched structure shaped by the river flow. Microorganisms are transferred from the water column to the underlying sediments and enrich them (Brown et al., 2011; De Oliveira, Margis, 2015; Mansour et al., 2018; Wang L. et al., 2018). The branched structure of the river ecosystem contributes to accumulation of bacteria from surrounding lands, including urban and industrial areas, wastewater treatment plants and agricultural lands, which also contain soluble components (Mansour et al., 2018). These include organic matter, nutrients or toxic compounds, and metals, which affect the activity and abundance of heterotrophic bacteria in bottom sediments (Fischer et al., 2002).

Microorganisms are crucial for energy conversion, biogeochemical nutrient cycling, pollutant degradation, and biotransformation of organic matter; therefore, bacteria can be used as bioindicators of aquatic ecosystems (Wei et al., 2008; Chen et al., 2018). For example, the abundance of Nitrospirae, Betaproteobacteriales, Chloroflexi, and Sphingobacteriales represantatives was found to increase in proportion to an increase in the concentration of nitrogen, which shows high concentrations due to anthropogenic load. An increased proportion of Nitrospirae, Sphingobacteriales (Bacteroidetes) and Spirochaetes and a generally decreased abundance of Actinobacteria were observed in sediment communities of river ecosystems located near wastewater treatment plants, which indicates the impact of wastewater (Sagova-Mareckova et al., 2021).

Thus, characterization of the composition of bacterial communities in the water column and river sediments, as well as the response of microbial communities to environmental changes, can yield valuable information to explore microbial interrelations and assess the environmental risk (Psenner et al., 2008; Wang J. et al., 2016). In addition, bottom sediments can be a source of metabolically diverse microorganisms, including those promising for industrial biotechnologies.

Works that address the species composition and functions of microbial communities in river ecosystems are few in number as compared, for example, with those related to ecosystems of salt lakes or seas. Microbiological studies of rivers flowing through the territory of Russia cover mainly their sanitary and epidemiological status (Shornikova, 2008). A.I. Kopylov and D.B. Kosolapov investigated distribution of bacterioplankton in the lower reaches of the Ob River and provided measurements of the specific growth rate, and the abundance and distribution of biomass in different parts of the river (Kopylov, Kosolapov, 2011). Other works related to microbiological monitoring of the Ob River studied the abundance and distribution of some metabolic groups of microorganisms (Savichev et al., 2015), including those resistant to antibiotics and phenol (Shornikova, Arslanova, 2020). Yet the species diversity and physiological characteristics of the native microflora have been poorly studied.

The Ob River flows through the territory of Western Siberia and ranks among the first in terms of length, water content and catchment area among Eurasian rivers. In the Siberian region, the Ob River is exposed to the greatest anthropogenic load, including demographic, agricultural and industrial impact; its water quality indicators for the content of certain metals and oil products are considered critical (Koronkevich et al., 2019). Therefore, the study of microbial communities in the water column and bottom sediments is of relevance, including the search for biotechnologically promising microorganisms – decomposers of organic matter

The aim of this study was to isolate pure cultures of microorganisms-decomposers from bottom sediments of the Ob River, analyze their ability to utilize various organic substrates, and detect their lipolytic and hydrocarbonoxidizing activity in different cultivation conditions.

## Materials and methods

Bottom sediment samples were collected in July 2020 in the middle reaches of the Ob River near the following settlements: Molchanovo (57.601429° N, 83.7824851° E), Kolpashevo (58.30456° N, 82.90774° E), Kargasok (59.06722° N, 80.84963° E). Sediments (sandy deposits) sampled from a depth of 1.5 m were put into sterile plastic test tubes and stored at +4 °C. The pH level of the water at the sampling sites was shifted to slightly alkaline pH values (from 7.5 to 8.6) (Frank et al., 2021).

Strains – decomposers of organic matter and producers of biotechnologically significant enzymes – were isolated by selective cultivation on culture media for lipolytic and hydrocarbon-oxidizing microorganisms. Initial enrichment cultures from each sampling site were obtained on a selective mineral medium containing pork fat (1 % of the medium volume) used as the only carbon source (Gerasimchuk et al., 2020) and on the medium used for hydrocarbon-oxidizing bacteria supplemented with 1 % diesel fuel, as described in (Frank et al., 2020). Samples taken in an amount of 0.5 ml from each site were inoculated in 50 ml of the liquid medium (pH 7.5) containing pork fat in 120 ml glass vials and cultivated at +28 °C in oxygen. The first inoculation to obtain hydrocarbon-oxidizing microorganisms was performed by limiting dilutions in 7 ml of the liquid medium in 15 ml glass penicillin vials. After that, the resulting enrichment cultures were inoculated on agar media of similar composition to obtain individual colonies. The colonies grown on plates with enrichment cultures were transferred to GRM broth (8 g/l pancreatic hydrolyzate of fish meal, 8 g/l enzymatic peptone, 4 g/l sodium chloride, pH 7.0–7.4). Then, the obtained aerobic strains were cultivated on Petri dishes with GRM broth at +28 °C.

The morphology of enrichment and pure cultures was analyzed by phase contrast microscopy (Biomed 6, Russia) using ×100 immersion lens.

The ability of strains to oxidize petroleum products was estimated using a liquid culture medium (g/l: KH2PO4 – 1.5, K2HPO4 – 0.75, NH4Cl – 1.0, NaCl – 2.5, MgSO4 · 7H2O – 0.2, yeast extract – 0.5) supplemented with 1 % diesel fuel as an organic substrate. The inoculations were performed in 15 ml penicillin vials filled with the medium to 1/3 (5 ml). Incubation proceeded at +28 °C. The growth was assessed by medium turbidity and using microscopy. To confirm the hydrocarbonoxidizing activity of individual strains, inoculations of the dense mineral medium without yeast extract (Gerasimchuk et al., 2020) were performed on Petri dishes with the addition of 0.1 ml of diesel fuel spread together with the inoculum on the medium surface.

The ability to utilize animal and vegetable fat was studied using a mineral culture medium (Gerasimchuk et al., 2020) containing 1 % pork fat or 1 % olive oil. The cultivation procedure was as follows: 0.25 ml of molten sterile pork fat or olive oil was spread on Petri dishes filled with 25 ml of the agar mineral medium; the inoculum was placed in a droplet of pork fat and spread on the surface of the medium using a spatula or a bacterial loop.

Lipolytic activity was detected using a diagnostic medium (tributyrin agar) containing 0.5 % (w/v) peptone, 0.3 % (w/v) yeast extract and 1.5 % bacteriological agar (pH 7.0) supplemented with 1 % tributyrin (Ramnath et al., 2017). Tributyrin is an ester composed of butyric acid and glycerol. Tributyrin agar is mainly used to detect lipolytic activity in bacteria (Mourey, Kilbertus, 1976). Cultures were incubated at +28, +25, and +4 °C. After 24 or 48 h of incubation, hydrolysis zones (transparent halos) could be observed around the colonies.

The phylogenetic position of the obtained strains was determined by sequencing and analyzing 16S rRNA gene sequences. Genomic DNA was isolated from cultures using the Biolabmix kit (DU-50) in accordance with the manufacturer’s recommendations (http://biolabmix.ru/). For amplification of bacterial 16S rRNA genes, which are universal phylogenetic markers, primers 27F (DeLong, 1992) and 1492R (Weisburg et al., 1991) were used. A 50 μl PCR mixture contained 1x PCR buffer (Biolabmix), 2.5 mM MgCl2 (Biolabmix), 0.2 mM dNTP mixture (Biolabmix), 10 pM of each primer (Sintol), 0.7 U thermostable HS-Taq polymerase (Biolabmix), 3 μl of template DNA (at a concentration exceeding 50 ng); the mixture was brought to the final volume with sterile deionized water.

The 16S rRNA genes were amplified in accordance with the procedure described in (Gerasimchuk et al., 2010). Sequencing of the obtained DNA sequences was performed using a genetic analyzer NANOFOR-05 in Scientific and Production Company “Sintol”, Moscow. To obtain a nearly complete 16S rRNA gene sequence, the forward primer 27F (DeLong, 1992), the reverse primer 1492R (Weisburg et al., 1991), and the BacV3F primer (Muyzer et al., 1993) were used.

The obtained nucleotide sequences of the 16S rRNA genes were analyzed using the BIOEDIT sequence editor (http:// www.jwbrown.mbio.ncsu.edu), the BLAST program in the NCBI GenBank database (http://www.ncbi.nlm.nih.gov), and the SILVA database classifier (http://www.arb-silva/aligner/ de). Chimeric sequences were detected using the DECIPHER package (http://www2.decipher.codes/FindChimeras.html). The obtained nucleotide sequences of the 16S rRNA gene fragments were deposited in the GenBank database under the numbers: OM212652, OM212653, OM212656–OM212659, OM212664–OM212671.

## Results and discussion

Enrichment and isolation of pure cultures

For isolation of bacterial strains – producers and decomposers of organic matter – samples of bottom sediments from the middle reaches of the Ob River and selective culture media for lipophilic and hydrocarbon-oxidizing microorganisms were used. Morphologically homogeneous pure cultures were isolated from enrichment cultures, which showed high abundance and morphological diversity of cell forms (see the Figure). Strains that exhibited stable growth on GRM agar were used for further studies. In total, 9 pure cultures were obtained from separate colonies isolated on the medium used for hydrocarbon-oxidizing bacteria, and 12 pure cultures were isolated on the medium used for lipolytics.

**Fig. 1.**
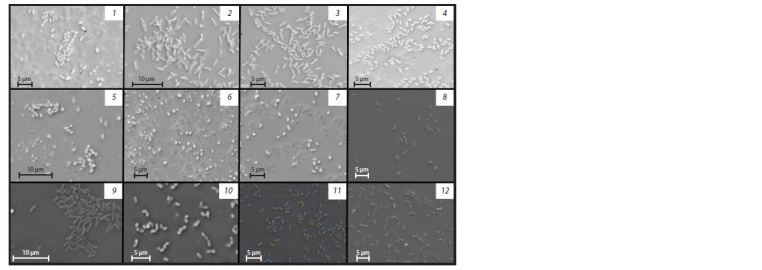
Micrographs of cells in the pure cultures: 1, LM7 strain; 2, LM6 strain; 3, LM8 strain; 4, KGS3Ps1 strain; 5, LKol1 strain; 6, LM3 strain; 7, LM4 strain; 8, KGS5k2 strain; 9, KGS3Ps2 strain; 10, KGS5k3 strain; 11, KGS5k1 strain; 12, LKol3 strain. Phase contrast microscopy, ×1000 magnification.

Phylogenetic analysis

Analysis of the 16S rRNA gene fragments showed that the strains belong to Proteobacteria (Gammaproteobacteria and Betaproteobacteria) (see the Table). Proteobacteria often dominate in the bacterial communities in water and bottom sediments, and their proportion in sediments is typically higher than that in water (Dai et al., 2016; Zhang et al., 2019).

Most of the analyzed strains were representatives of Pseudomonas and Aeromonas (Gammaproteobacteria). All the obtained and analyzed fragments of the 677–1445 bp DNA sequences showed a high percentage of similarity (99.48– 100 %) with sequences of typical strains of microorganisms deposited in the GenBank NCBI database.

A part of the strains was related to opportunistic pathogens belonging to hazard group II according to the WHO classification (https://bacdive.dsmz.de/). The detected pathogens included all Aeromonas strains related to A. veronii (LKar2 and LKar3), A. hydrophila (LM7 and KLP3), as well as E. coli (LKol1) and P. putida (LM3 and LM4). Most of the pathogenic strains were isolated from lipophilic enrichment cultures. Conditionally pathogenic microorganisms (enterobacteria related to Serratia marcescens, Leclercia adecarboxylata, Klebsiella pneumoniae, K. huaxiensis, Enterobacter cloacae, Raoultella ornithinolytica, Morganella morganii) grown on the mineral medium containing pork fat were isolated in our previous studies when isolating pure cultures of lipophilic microorganisms from wastewater treatment plant effluent and food industry wastewater (Gerasimchuk et al., 2020). Bacterial lipases involved in such metabolic processes as hydrolysis and lipid modification can be virulence factors for some phylogenetic groups, which explains numerous pathogens found among lipophilic bacteria (Bender, Flieger, 2010; Kovacic et al., 2019).

Other bacteria were related to microorganisms that are nonhuman pathogens. Most of the strains were representatives of the genus Pseudomonas. The genus Pseudomonas includes a large group of Gram-negative bacteria that exhibit a great metabolic diversity, which allows them to utilize a wide range of organic compounds and play important ecological roles in the carbon cycling. Pseudomonas are ubiquitous in a wide variety of ecosystems and include many pathogenic human, animal, and plant species (Peix et al., 2009), as well as mutualistic species, which include the most remarkable representatives of biocontrol strains that protect plants from pathogens (Ramette et al., 2011; De Vrieze et al., 2015). Pseudomonades can degrade various lipids and lipidcontaining compounds (Pabai et al., 1996; Lee, Rhee, 2008; Yang J. et al., 2009; Fendri et al., 2010), as well as oil hydrocarbons (Barathi, Vasudevan, 2001). Representatives of the genus Pseudomonas are often found in river ecosystems, which is evidenced by molecular studies (Cyriaque et al., 2020) and cultural methods (Pellett et al., 1983; Pirnay et al., 2005), including isolation of new pseudomonades from oil-contaminated bottom sediments in China (Li et al., 2020), dioxin-contaminated bottom sediments in Texas (Iyer et al., 2017), bottom sediments in India (Sudan et al., 2018), etc.

Analysis of the 16S rRNA gene sequencing performed for the Mol4a strain showed 100 percentage of similarity with sequences of P. veronii strains from various habitats (activated sludge, hydrocarbon-contaminated groundwater, contaminated sediments, etc.), and 99.82 percentage of similarity with the P. veronii type strain isolated from mineral waters (Elomari et al., 1996). The KGS3Ps2 strain was found to be closely related to P. protegens (LS999205) isolated from soil, which together with representatives of P. veronii belongs to the Pseudomonas fluorescens group. P. baetica belongs to the same taxonomic group, and its 16S rRNA gene showed 100 percentage of sequence similarity with the KGS3Ps1 strain. The type strain of the above bacterium is a pathogen for sole (López et al., 2012).

The KGS5k1 strain showed the highest percentage of similarity (99.86 %) with an undescribed strain isolated from soil and referred to as P. brassicacearum (KT695825), and 99.5 percentage of similarity with a valid strain P. chlororaphis (CP027720) isolated from fluviatile loam and related to the Pseudomonas chlororaphis taxonomic group. Comparison of the nearly complete 16S rRNA gene sequence showed a similar percentage of similarity between the Mol4k12 strain and the type strains of different species, namely, P. fildesensis (MK859934) and P. extremaustralis (KX186942), which are closely related to representatives of P. fluorescens. KGS5k2, KGS5k3, and KGS5k8 strains showed 100 % homology with the 16S rRNA gene of P. lini (NR_029042) isolated from rhizosphere soil (Delorme et al., 2002).

The genus Pseudomonas is one of the taxonomically most complex genera (Parte, 2014). Although the 16S rRNA gene is a universal phylogenetic marker in the bacterial classification system, the analysis of this gene alone does not allow differentiation of closely related bacterial species. Recent studies have shown that multilocus sequence analysis (MLSA) performed for four housekeeping genes (16S rRNA, gyrB, rpoB, and rpoD) enables species identification and facilitates strain identification in Pseudomonas (Mulet et al., 2012). Thus, a more precise determination of the phylogenetic position of the isolated Pseudomonas strains requires analysis of additional molecular markers (for example, gyrB and rpoD genes).

Analysis of 16S rRNA gene sequences showed that LM1, LM2, LM6, LM8, LKol2, and LKol3 strains are identical and can be assigned to the genus Microvirgula, the class Betaproteobacteria. Interestingly, we earlier isolated the identical 768 bp sequence (GenBank accession number MT476921) that belong to bacteria of the genus Microvirgula from food industry waste (Gerasimchuk et al., 2020). Representatives of the genus Microvirgula grow well in aerobic and anaerobic conditions, have an atypical respiratory type of metabolism, and use oxygen and nitrogen oxides as the final electron acceptors (Patureau et al., 1998). The genus Microvirgula was first described by Patureau et al. (1998) and was characterized as a new denitrifying bacterium M. aerodenitrificans isolated from activated sludge. At present, the genus Microvirgula includes two species. The other representative of M. curvata was isolated from hydrocarboncontaminated soil (Subhash et al., 2016) and included one strain. Comparison of the sequenced fragments of the 16S rRNA genes of LM1, LM2, LM6, LM8, LKol2, and LKol3 strains showed that their sequences are identical and exhibit 100 % homology with the strain of M. aerodenitrificans (MT367755) isolated from the intestines of wild animals. They also showed 99.79 and 99.86 percentage of similarity with M. aerodenitrificans Sgly2 isolated from activated sludge (Patureau et al., 1998) and the type strain, M. aerodenitrificans NBRC 15328 (AB680837) isolated from fresh water (Cleenwerck et al., 2003), respectively.

Lipolytic activity of numerous representatives of Pseudomonas was thoroughly investigated on various substrates, lipolytic enzyme genes were studied and cloned (Reetz, Jaeger, 1998; Bofill et al., 2010; Yang W. et al., 2015; Cai et al., 2016), and their hydrocarbon-oxidizing activity was confirmed (Muriel-Millán et al., 2019). At the same time, only lipolytic activity on diagnostic media was shown for representatives of the genus Microvirgula, and their lipolytic properties were not studied in detail. Yet the analysis of Microvirgula genomes available in the NCBI database revealed lipolytic enzyme genes. At present, data have been published on the genomes of two strains of M. aerodenitrificans (JHVK01000000 and CP028519) isolated from different bioreactors and one strain of Microvirgula (NZ_QLTJ01000000) with an unidentified phylogenetic position, which is a bacterial endophyte of rice. The search for lipolytic enzyme genes in the listed genomes revealed the presence of lipases and esterases. Additionally, data on sequences of Microvirgula strains isolated from petroleum-contaminated habitats (the GenBank database, accession numbers KM357844, LT631813) indirectly indicate their hydrocarbon-oxidizing activity.

Detection of lipolytic activity of strains using a diagnostic medium

Non-pathogenic strains of different phylogenetic affiliation with nearly complete 16S rRNA sequences (see the Table) and different growth or morphology characteristics were used to study lipolytic activity. All the strains grew and formed hydrolysis zones on tributyrin agar after 24–48 h of cultiva-tion at +28 °C, except for P. veronii sp. Mol4, which did not form hydrolysis zones, most likely due to the absence of lipolytic activity and the use of peptone, the component of the culture medium, as a growth substrate. In contrast to other strains with hydrolysis zones of about 3 mm, P. protegens sp. KGS3Ps2, P. brassicacearum sp. KGS5k1, and M. aerodenitrificans sp. LM1 showed a more pronounced lipolytic activity in the form of complete hydrolysis reaction. Additionally, P. protegens sp. KGS3Ps2 and P. brassicacearum sp. KGS5k1 exhibited growth and lipolytic activity at +4 °C.

**Table 1.**
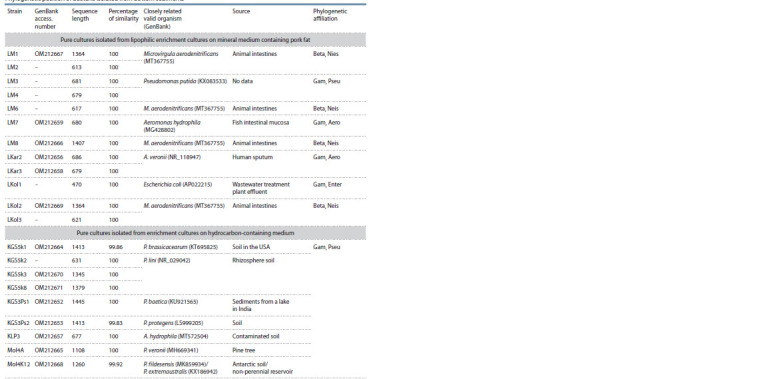
Phylogenetic position of bacteria isolated from bottom sediments Note. Beta, Nies – Betaproteobacteria, Neisseriales; Gam, Pseu – Gammaproteobacteria, Pseudomonadales; Gam, Enter – Gammaproteobacteria, Enterobacterales; Gam, Aero – Gammaproteobacteria, Aeromonadales.

Study of the ability of strains to utilize organic substrates

In contrast to Microvirgula strains, Pseudomonas strains grown on GRM agar exhibited psychrotolerant properties and stable growth at +4 °C. None of the studied thermotolerant strains showed stable growth at +50 °C. There were no significant difference in biomass gain on GRM and tributyrin agar at +25 and +28 °C.

On dense media containing 1 % pork fat and 1 % olive oil, strains of M. aerodenitrificans sp. LM1 and P. lini sp. KGS5k3 were observed to grow at +25 and +28 °С. The inoculations on media containing pork fat and olive oil at +4 °C showed that animal fat and vegetable oil do not degrade or this process is constrained at low temperatures. P. protegens sp. KGS3Ps2 and P. brassicacearum sp. KGS5k1 did not grow on these media even at +28 °C despite their more pronounced lipolytic activity on diagnostic media

Screening of strains on the selective medium containing 1 % diesel fuel showed that 5 out of 10 strains, namely, P. protegens sp. KGS3Ps2, M. aerodenitrificans sp. LM1, P. fildesensis/ extremaustralis sp. Mol4K12, and P. lini spp. KGS5k3 and KGS5k8, are able to grow on a hydrocarbon-containing medium. To confirm the hydrocarbon-oxidizing activity of M. aerodenitrificans sp. LM1 and P. lini sp. KGS5k3 – the most promising decomposers – additional inoculations were carried out on a dense mineral medium containing diesel fuel as the only carbon source. The growth of strains was observed after less than 2 days. It should be noted that P. lini sp. KGS5k3 yielded more biomass.

## Conclusion

The phylogenetic and metabolic diversity of cultivated nonpathogenic bacterial strains with lipolytic and hydrocarbonoxidizing activity revealed in the study indicates biotechnological potential of the isolates. The most promising strains are M. aerodenitrificans sp. LM1 and P. lini sp. KGS5k3, which exhibited lipolytic activity on a diagnostic medium in a wide temperature range and utilized such complex organic substrates as diesel fuel, pork fat and olive oil. For the first time, the ability to oxidize petroleum products and grow on specific fat-containing substrates was shown for representatives of M. aerodenitrificans. Earlier, only lipolytic activity on diagnostic media was reported for M. aerodenitrificans (Patureau et al., 1998). The biotechnological potential of M. aerodenitrificans described in the literature indicates its ability for aerobic and anaerobic denitrification in waste treatment technologies using bioreactors (Patureau et al., 2001; Bouchez et al., 2009; Anderson et al., 2020). However, isolation of 16S rRNA phylotypes and pure cultures related to the genus Microvirgula from hydrocarbon-contaminated samples (Subhash et al., 2016; Sarkar et al., 2017) and waste effluents (Cea et al., 2015; Gerasimchuk et al., 2020), and our results on the growth obtained using media containing fat and oil products, indicate a wider biotechnological potential of these microorganisms.

No literature data were found on lipolytic activity of P. lini. Thus, we have shown for the first time lipolytic activity of representatives of this species using a diagnostic medium, and their ability to utilize oil products and animal fat.

## Conflict of interest

The authors declare no conflict of interest.

## References

Anderson E.L., Jang J., Venterea R.T., Feyereisen G.W., Ishii S.
Isolation and characterization of denitrifiers from woodchip
bioreactors for bioaugmentation application. J. Appl. Microbiol.
2020;129(3):590-600. DOI 10.1111/jam.14655.

Araya R., Tani K., Takagi T., Yamaguchi N., Nasu M. Bacterial
activity
and community composition in stream water and
biofilm from an urban river determined by fluorescent in situ
hybridization and DGGE analysis. FEMS Microbiol. Ecol.
2003;43(1):111-119. DOI 10.1111/j.15746941.2003.tb01050.x.

Barathi S., Vasudevan N. Utilization of petroleum hydrocarbons
by Pseudomonas f luorescens isolated from petroleum contaminated
soil. Environ. Int. 2001;26:413-416. DOI 10.1016/S0160-
4120(01)00021-6.

Bender J., Flieger A. Lipases as pathogenicity factors of bacterial
pathogens of humans. In: Timmis K.N. (Ed.) Handbook of
Hydrocarbon and Lipid Microbiology. Berlin; Heidelberg:
Springer-Verlag, 2010:3241-3258. DOI 10.1007/978-3-540-
77587-4_246.

Bofill C., Prim N., Mormeneo M., Manresa A., Pastor F.I.J., Diaz P.
Differential behaviour of Pseudomonas sp. 42A2 LipC, a lipase
showing greater versatility than its counterpart LipA. Biochimie.
2010;92(3):307-316. DOI 10.1016/j.biochi.2009.11.005

Bouchez T., Patureau D., Delgenès J.P., Moletta R. Successful
bacterial incorporation into activated sludge flocs using alginate.
Bioresour. Technol. 2009;100(2):1031-1032. DOI 10.1016/
j.biortech.2008.07.028.

Brown B.L., Swan C.M., Auerbach D., Campbell Grant E.H.,
Hitt N.P., Maloney K.O., Patrick C. Metacommunity theory as
a multispecies, multiscale framework for studying the influence
of river network structure on riverine communities and ecosystems.
J. North Am. Benthol. Soc. 2011;30(1):310-327. DOI
10.1899/10-129.1.

Cai X., Chen S., Yang H., Wang W., Lin L., Shen Y., Wei D. Biodegradation
of waste greases and biochemical properties of a novel
lipase from Pseudomonas synxantha PS1. Can. J. Microbiol.
2016;62(7):588-599. DOI 10.1139/cjm-2015-0641.

Cea M., Sangaletti-Gerhard N., Acuña P., Fuentes I., Jorquera M.,
Godoy K., Osses F., Navia R. Screening transesterifiable lipid
accumulating bacteria from sewage sludge for biodiesel production.
Biotechnol. Rep. 2015;8:116-123. DOI 10.1016/
j.btre.2015.10.008.

Chen J., Wang P.F., Wang C., Wang X., Miao L.Z., Liu S., Yuan Q.S.
Bacterial communities in riparian sediments: a large-scale longitudinal
distribution pattern and response to dam construction.
Front. Microbiol. 2018;9:999. DOI 10.3389/fmicb.2018.00999

Cleenwerck I., De Wachter M., Hoste B., Janssens D., Swings J.
Aquaspirillum dispar Hylemon et al. 1973 and Microvirgula
aerodenitrificans Patureau et al. 1998 are subjective synonyms.
Int. J. Syst. Evol. Microbiol. 2003;53(5):1457-1459. DOI 10.1099/
ijs.0.02675-0.

Cyriaque V., Géron A., Billon G., Nesme J., Werner J., Gillan D.C.,
Wattiez R. Metal-induced bacterial interactions promote diversity
in river-sediment microbiomes. FEMS Microbiol. Ecol.
2020;96(6):5826176. DOI 10.1093/femsec/fiaa076.

Dai Y., Yang Y.Y., Wu Z., Feng Q.Y., Xie S.G., Liu Y. Spatiotemporal
variation of planktonic and sediment bacterial assemblages
in two plateau freshwater lakes at different trophic status. Appl.
Microbiol. Biotechnol. 2016;100(9):4161-4175. DOI 10.1007/
s00253-015-7253-2.

DeLong E.F. Archaea in costal marine environments. Proc.
Natl. Acad. Sci. USA. 1992;89:5685-5689. DOI 10.1073/
pnas.89.12.5685

Delorme S., Lemanceau P., Christen R., Corberand T., Meyer
J.M., Gardan L. Pseudomonas lini sp. nov., a novel species
from bulk and rhizospheric soils. Int. J. Syst. Evol. Microbiol.
2002;52(2):513-523. DOI 10.1099/00207713-52-2-513.

de Oliveira L.F.V., Margis R. The source of the river as a nursery
for microbial diversity. PLoS One. 2015;10(3):e0120608. DOI
10.1371/journal.pone.0120608.

De Vrieze M., Pandey P., Bucheli T.D., Varadarajan A.R.,
Ahrens C.H., Weisskopf L., Bailly A. Volatile organic compounds
from native potato-associated Pseudomonas as potential
anti-oomycete agents. Front. Microbiol. 2015;6:1295. DOI
10.3389/fmicb.2015.01295.

Elomari M., Coroler L., Hoste B., Gillis M., Izard D., Leclerc H.
DNA relatedness among Pseudomonas strains isolated from
natural mineral waters and proposal of Pseudomonas veronii
sp. nov. Int. J. Syst. Bacteriol. 1996;46(4):1138-1144. DOI
10.1099/00207713-46-4-1138.

Fendri I., Chaari A., Dhouib A., Jlassi B., Abousalham A., Carrière
F., Sayadi S., Abdelkafi S. Isolation, identification and
characterization of a new lipolytic Pseudomonas sp., from
Tunisian soil. Environ. Technol. 2010;31(1):87-95. DOI
10.1080/09593330903369994.

Fischer H., Wanner S.C., Pusch M. Bacterial abundance and production
in river sediments as related to the biochemical composition
of particulate organic matter (POM). Biogeochemistry.
2002;61:37-55. DOI 10.1023/A:1020298907014.

Frank Y.A., Nikitchuk K.L., Sapega A.A., Lukjanova E.A.,
Ivasenko D.A., Kosov A.V., Gerasimchuk A.L., Evseeva N.S.
Improvement of the efficiency of oil-contaminated soils remediation
in the natural conditions of the north Tomsk region and
the nearby regions by indigenous microorganisms application.
Izvestiya Tomskogo Polytehnicheskogo Universita. Inzhiniring
Georesursov = Bulletin of the Tomsk Polytechnic University.
Geo Аssets Engineering. 2020;331(9):130-139. DOI
10.18799/24131830/2020/9/2815. (in Russian)

Frank Y.A., Vorobiev E.D., Vorobiev D.S., Trifonov A.A., Antsiferov
D.V., Soliman Hunter T., Wilson S.P., Strezov V. Preliminary
screening for microplastic concentrations in the surface
water of the Ob and Tom rivers in Siberia, Russia. Sustainability.
2021;13(1):80. DOI 10.3390/su13010080.

Gerasimchuk A.L., Ivasenko D.A., Bukhtiyarova P.A., Antsiferov
D.V., Frank Y.A. Search for new cultured lipophilic bacteria
in industrial fat-containing wastes. BIO Web Conf. II Int.
Sci. Conf. “Plants and Microbes: The Future of Biotechnology”
(PLAMIC2020). 2020;23:02012. DOI 10.1051/bioconf/
20202302012.

Gerasimchuk A.L., Shatalov A.A., Novikov A.L., Butorova O.P.,
Pimenov N.V., Lein A.Y., Yanenko A.S., Karnachuk O.V. The
search for sulfate-reducing bacteria in mat samples from the lost
city hydrothermal field by molecular cloning. Microbiology.
2010;79(1):96-105. DOI 10.1134/S0026261710010133.

Iyer R., Iken B., Damania A. Genome of Pseudomonas nitroreducens
DF05 from dioxin contaminated sediment downstream of
the San Jacinto River waste pits reveals a broad array of aromatic
degradation gene determinants. Genom. Data. 2017;17(14):40-
43. DOI 10.1016/j.gdata.2017.07.011.

Kopylov A.I., Kosolapov D.B. The structure of the planktic microbial
community in the lower reaches of the Ob river near
Salekhard. Contemp. Probl. Ecol. 2011;4(1):1-7. DOI 10.1134/
S1995425511010012.

Koronkevich N.I., Barabanova E.A., Georgiadi A.G., Zaitseva
I.S., Shaporenko S.I. Anthropogenic impacts on the water resources of the Russian Arctic basin rivers. Geogr. Nat. Resour.
2019;40(1):22-29. DOI 10.1134/S1875372819010049.

Kovacic F., Babić N., Krauss U., Jaeger K.-E. Classification of lipolytic
enzymes from bacteria. In: Rojo F. (Ed.) Aerobic Utilization
of Hydrocarbons,
Oils, and Lipids. Handbook of hydrocarbon
and lipid microbiology. Cham: Springer, 2019;255-289.
DOI 10.1007/978-3-319-50418-6_39.

Lee S.Y., Rhee J.S. Hydrolysis of triglyceride by the whole cell
of Pseudomonas putida 3SK in two-phase batch and continuous
reactors systems. Biotechnol. Bioeng. 2008;44:437-443. DOI
10.1002/bit.260440406.

Li J., Wang L.-H., Xiang F.-G., Ding W.-L., Xi L.-J., Wang M.-Q.,
Xiao Z.-J., Liu J.-G. Pseudomonas phragmitis sp. nov., isolated
from petroleum polluted river sediment. Int. J. Syst. Evol. Microbiol.
2020;70(1):364-372. DOI 10.1099/ijsem.0.003763.

López J.R., Diéguez A.L., Doce A., De la Roca E., De la Herran R.,
Navas J.I., Toranzo A.E., Romalde J.L. Pseudomonas baetica
sp. nov., a fish pathogen isolated from wedge sole, Dicologlossa
cuneata (Moreau). Int. J. Syst. Evol. Microbiol. 2012;62(4):874-
882. DOI 10.1099/ijs.0.030601-0

Mansour I., Heppell C.M., Ryo M., Rillig M.C. Application of the
microbial community coalescence concept to riverine networks.
Biol. Rev. 2018;93(4):1832-1845. DOI 10.1111/brv.12422.

Mourey A., Kilbertus G. Simple media containing stabilized
tributyrin for demonstrating lipolytic bacteria in foods and
soils. J. Appl. Bacteriol. 1976;40:47-51. DOI 10.1111/j.1365-
2672.1976.tb00589.x.

Mulet M., Gomila M., Lemaitre B., Lalucat J., García-Valdés E.
Taxonomic characterization of Pseudomonas strain L48
and formal proposal of Pseudomonas entomophila sp. nov.
Syst. Appl. Microbiol. 2012;35:145-149. DOI 10.1016/
j.syapm.2011.12.003.

Muriel-Millán L.F., Rodríguez-Mejía J.L., Godoy-Lozano E.E.,
Rivera-Gómez N., Gutierrez-Rios R.-M., Morales-Guzmán D.,
Trejo-Hernández M.R., Estradas-Romero A., Pardo-López L.
Functional and genomic characterization of a Pseudomonas
aeruginosa strain isolated from the southwestern gulf of
Mexico reveals an enhanced adaptation for long-chain alkane
degradation. Front. Mar. Sci. 2019;6:572. DOI 10.3389/
fmars.2019.00572.

Muyzer G., de Waal E.C., Uitterlinden U.G. Profiling of complex
microbial populations by denaturing gradient gel electrophoresis
analysis of polymerase chain reaction-amplified genes coding
for 16S rRNA. Appl. Environ. Microbiol. 1993;59(3):695-700.
DOI 10.1128/aem.59.3.695-700.1993.

Pabai F., Kermasha S., Morin A. Use of continuous culture to screen
for lipase-producing microorganisms and interesterification of
butterfat by lipase isolates. Can. J. Microbiol. 1996;42:446-452.
DOI 10.1139/m96-061.

Parte A. LPSN-list of prokaryotic names with standing in nomenclature.
Nucleic Acids Res. 2014;42:D613-D616. DOI 10.1093/
nar/gkt1111.

Patureau D., Godon J.J., Dabert P., Bouchez T., Bernet N.,
Delgenes J.P., Moletta R. Microvirgula aerodenitrificans gen.
nov., sp. nov., a new gram-negative bacterium exhibiting corespiration
of oxygen and nitrogen oxides up to oxygen-saturated
conditions. Int. J. Syst. Bacteriol. 1998;48:775-782. DOI
10.1099/00207713-48-3-775.

Patureau D., Helloin E., Rustrian E., Bouchez T., Delgene J.,
Moletta R. Combined phosphate and nitrogen removal in a sequencing
batch reactor using the aerobic denitrifier, Microvirgula
aerodenitrificans. Water Res. 2001;35(1):189-197. DOI
10.1016/s0043-1354(00)00244.

Peix A., Ramírez-Bahena M.-H., Velázquez E. Historical evolution
and current status of the taxonomy of genus Pseudomonas.
Infect. Genet. Evol. 2009;9(6):1132-1147. DOI 10.1016/j.
meegid.2009.08.001.

Pellett S., Bigley V.D., Grimes D.J. Distribution of Pseudomonas
aeruginosa
in a riverine ecosystemt. Appl. Environ. Microbiol.
1983;45(1):328-332. DOI 10.1128/aem.45.1.328-332.1983.

Pirnay J.-P., Matthijs S., Colak H., Chablain P., Bilocq F., Van Eldere J.,
De Vos D., Zizi M., Triest L., Cornelis P. Global Pseudomonas
aeruginosa
biodiversity as reflected in a Belgian river. Environ.
Microbiol. 2005;7(7):969-980. DOI 10.1111/j.1462-2920.2005.
00776.x.

Psenner R., Alfreider A., Schwarz A. Aquatic microbial ecology:
water desert, microcosm, ecosystem. What’s тext?
Internat. Rev. Hydrobiol. 2008;93(4-5):606-623. DOI 10.1002/
IROH.200711044

Ramette A., Frapolli M., Saux M.F.-L., Gruffaz C., Meyer J.-M.,
Défago G., Sutra L., Moënne-Loccoz Y. Pseudomonas protegens
sp. nov., widespread plant-protecting bacteria producing
the biocontrol compounds 2,4-diacetylphloroglucinol and pyoluteorin.
Syst. Appl. Microbiol.
2011;34(3):180-188. DOI 10.1016/
j.syapm.2010.10.005.

Ramnath L., Sithole B., Govinden R. Identification of lipolytic
enzymes isolated from bacteria indigenous to Eucalyptus wood
species for application in the pulping industry. Biotechnol. Rep.
2017;15:114-124. DOI 10.1016/j.btre.2017.07.004.

Reetz M.T., Jaeger K.E. Overexpression, immobilization and
biotechnological application of Pseudomonas lipases. Chem.
Phys. Lipids. 1998;93(1-2):3-14. DOI 10.1016/s0009-
3084(98)00033-4.

Sagova-Mareckova M., Boenigk J., Bouchez A., Cermakova K.,
Chonova T., Cordier T., Eisendle U., Elersek T., Fazi S.,
Fleituch T., Frühe L., Gajdosova M., Graupner N., Haegerbaeumer
A., Kelly A.-M., Kopecky J., Leese F., Nõges P.,
Orlic S., Panksep K., Pawlowski J., Petrusek A., Piggott J.J.,
Rusch J.C., Salis R., Schenk J., Simek K., Stovicek A.,
Strand D.A., Vasquez M.I., Vrålstad T., Zlatkovic S., Zupancic
M., Stoeck T. Expanding ecological assessment by integrating
microorganisms into routine freshwater biomonitoring. Water
Res. 2021;191:116767. DOI 10.1016/j.watres.2020.116767.

Sarkar P., Roy A., Pal S., Mohapatra B., Kazy S.K., Maiti M.K.,
Sar P. Enrichment and characterization of hydrocarbon-degrading
bacteria from petroleum refinery waste as potent bioaugmentation
agent for in situ bioremediation. Bioresour. Technol.
2017;242:15-27. DOI 10.1016/j.biortech.2017.05.010.

Savichev O.G., Tokarenko O.G., Pasechnik E.Yu., Nalivaiko N.G.,
Ivanova Е.A., Nadeina L.V. Microbiological composition of
river waters in the Ob’ basin (West Siberia) and its associations
with hydrochemical indices. IOP Conf. Series: Earth Environ.
Sci. 2015;27:012035. DOI 10.1088/1755-1315/27/1/012035

Shornikova E.A. Microbiological indication of river ecosystem conditions
at the oil fields in the Middle Ob’ area. Contemp. Probl.
Ecol. 2008;1(3):328-334. DOI 10.1134/S1995425508030077.

Shornikova E., Arslanova M. The experience of application of
microbiological indicators in monitoring procedures of
aquatic ecosystems in the Middle Ob basin. E3S Web Conf.
2020;210:07013. DOI 10.1051/e3sconf/202021007013.

Subhash Y., Park M.J., Lee S.S. Microvirgula curvata sp. nov.,
isolated from hydrocarbon-contaminated soil, and emended description
of the genus Microvirgula. Int. J. Syst. Evol. Microbiol.
2016;66:5309-5313. DOI 10.1099/ijsem.0.001512.

Sudan S.K., Pal D., Bisht B., Kumar N., Chaudhry V., Patil P.,
Sahni G., Mayilraj S., Krishnamurthi S. Pseudomonas fluvialis sp. nov., a novel
member of the genus Pseudomonas isolated
from the river Ganges, India. Int. J. Syst. Evol. Microbiol.
2018;68(1):402-408. DOI 10.1099/ijsem.0.002520

Wang J., Li Y., Wang P., Niu L., Zhang W., Wang C. Response of
bacterial community compositions to different sources of pollutants
in sediments of a tributary of Taihu Lake, China. Environ.
Sci. Pollut. Res. Int. 2016;23(14):13886-13894. DOI 10.1007/
s11356-016-6573-9.

Wang L., Zhang J., Li H., Yang Н., Peng C., Peng Z., Lu L. Shift in
the microbial community composition of surface water and sediment
along an urban river. Sci. Total. Environ. 2018;627:600-
612. DOI 10.1016/j.scitotenv.2018.01.203.

Wei C.L., Bao S., Zhu X.Y., Huang X.X. Spatio-temporal variations
of the bacterioplankton community composition in
Chaohu Lake, China. Prog. Nat. Sci. 2008;18(9):1115-1122.
DOI 10.1016/j.pnsc.2008.04.005.

Weisburg W.G., Barns S.M., Pelletier D.A., Lane D.J. 16S ribosomal
DNA amplification for phylogenetic study. J. Bacteriol.
1991;173:697-703. DOI 10.1128/jb.173.2.697-703.1991.

Yang J., Zhang B., Yan Y. Cloning and expression of Pseudomonas
f
luorescens
26-2 lipase gene in Pichia pastoris and characterizing
for transesterification. Appl. Biochem. Biotechnol.
2009;159:355-365. DOI 10.1007/s12010-008-8419-5.

Yang W., Cao H., Xu L., Zhang H., Yan Y. A novel eurythermic
and thermostale lipase LipM from Pseudomonas moraviensis
M9 and its application in the partial hydrolysis of algal oil. BMC
Biotechnol. 2015;15:94. DOI 10.1186/s12896-015-0214-0.

Zhang L., Zhao T., Wang Q., Li L., Shen T., Gao G. Bacterial
community composition in aquatic and sediment samples with
spatiotemporal dynamics in large, shallow, eutrophic Lake
Chaohu, China. J. Freshw. Ecol. 2019;34(1):575-589. DOI
10.1080/02705060.2019.1635536.

